# The complete chloroplast genome of *Rubus pacificus* (Rosaceae)

**DOI:** 10.1080/23802359.2022.2116956

**Published:** 2022-09-08

**Authors:** Xian-Hua Xiong, Zheng-Qiao Liao, Chao Liu, Cheng Zhang, Xiong Li

**Affiliations:** a College of Life Science and Biotechnology, Mianyang Teachers’ College, Mianyang, China; b Chengdu Institute of Biology, Chinese Academy of Sciences, Chengdu, China; c College of Life Science, University of Chinese Academy of Sciences, Beijing, China

**Keywords:** *Rubus*, chloroplast genome, Rosaceae

## Abstract

The complete chloroplast genome of *Rubus pacificus*, a representative species of *R.* sect. *Malachobatus* subsect. *Stipulosi*, has been characterized by reference-based assembly using Illumina paired-end data. The complete chloroplast genome is 156,255 bp in length, containing a large single-copy region (LSC) of 85,864 bp and a small single-copy region (SSC) of 18,849 bp, which are separated by a pair of inverted repeat regions (IR) of 25,771 bp. A total of 129 genes were predicted from the chloroplast genome, including 85 protein-coding genes, 36 tRNA genes, and eight rRNA genes. According to the present sampling, phylogenetic analysis reveals that *R. pacificus* and *R. laciniatostipulatus* form a strongly supported clade and *R.* sect. *Malachobatus* subsect. *Stipulosi* is not monophyletic.

*Rubus* L. sect. *Malachobatus* Focke subsect. *Stipulosi* Yu and Lu (1982) contains approximately six species that occur in China. Morphologically, this subsection is characterized by shrubs, leaves simple, stipules and bracts free, broader, usually 2–5 × 1–2 cm. The genetic relationship of this subsection relative to other *Rubus* species is poorly understood. *Rubus pacificus* Hance (1874) is a representative species of *R.* sect. *Malachobatus* subsect. *Stipulosi*. In this study, we first reported the complete chloroplast genome of *R. pacificus* to examine the phylogenetic relationships of *R.* sect. *Malachobatus* subsect. *Stipulosi* and other *Rubus* species.

The plant material of *Rubus pacificus* was obtained from Lushan Mountain, Jiujiang, Jiangxi Province, China (29°39′0.20″ N, 116°3′58.10″ E, altitude 95 m). A specimen was deposited in the Herbarium of Chengdu Institute of Biology (CDBI, http://www.cib.ac.cn/, contact Xian-Hua Xiong, xianhua007@126.com) under the voucher number *X.H. Xiong 2262* and it was identified by Xian-Hua Xiong.

The total genomic DNA was extracted from fresh leaves using a modified CTAB method (Doyle and Doyle 1987) and sequenced based on the Illumina pair-end technology by Novogene Bioinformatics Technology Co., Ltd., Beijing, China. A total of 3.91 Gb clean reads were assembled using the programme GetOrganelle v1.7.5 (Jin et al. 2020) with the complete chloroplast genome of *Rubus crassifolius* as the reference (GenBank accession No. NC_056941). The assembled chloroplast genome was annotated using PGA (Qu et al. 2019), and the annotation result was modified using Geneious Prime 2020 (Biomatters Ltd., Auckland, New Zealand). Phylogenetic analysis including *R. pacificus*, 31 other *Rubus* species (with published plastid genomes) and two outgroups were performed using complete chloroplast genomes. The sequences were aligned using the MAFFT v7.490 (Katoh and Standley 2013). Poorly aligned regions were trimmed using Gblocks v.0.91b (Castresana 2000) with the parameters ‘–t = d − b4 = 5 − b5 = h.’ The maximum-likelihood (ML) tree was constructed using RAxML v.8.2.12 (Stamatakis 2014) with 1000 bootstrap replicates to examine the phylogenetic position of *R. pacificus* in genus *Rubus*.

The complete chloroplast genome of *Rubus pacificus* (Genbank accession No. ON243759) was a circular molecular genome with a size of 156,255 bp in length, which presented a typical quadripartite structure containing a pair of inverted repeat (IR) regions of 25,771 bp each, separated by the large single-copy (LSC) region of 85,864 bp, and small single-copy (SSC) region of 18,849 bp. The overall GC content was about 37.17%. The chloroplast genome consists of 129 genes including 85 protein-coding genes, 36 tRNA genes, and eight rRNA genes.

According to the present sampling, our phylogenetic result (Figure 1) showed that *Rubus pacificus* of *R.* sect. *Malachobatus* subsect. *Stipulosi* and *R. laciniatostipulatus* (*R. alceifolius*) of *R.* sect. *Malachobatus* subsect. *Moluccani* (Focke) Yu et Lu (1982) formed a strongly supported clade. And the two species of *R.* sect. *Malachobatus* subsect. *Stipulosi*—*R. pacificus* and *R. crassifolius* Yu and Lu (1982)—did not cluster together, suggesting that this subsection is not monophyletic.

**Figure 1.**
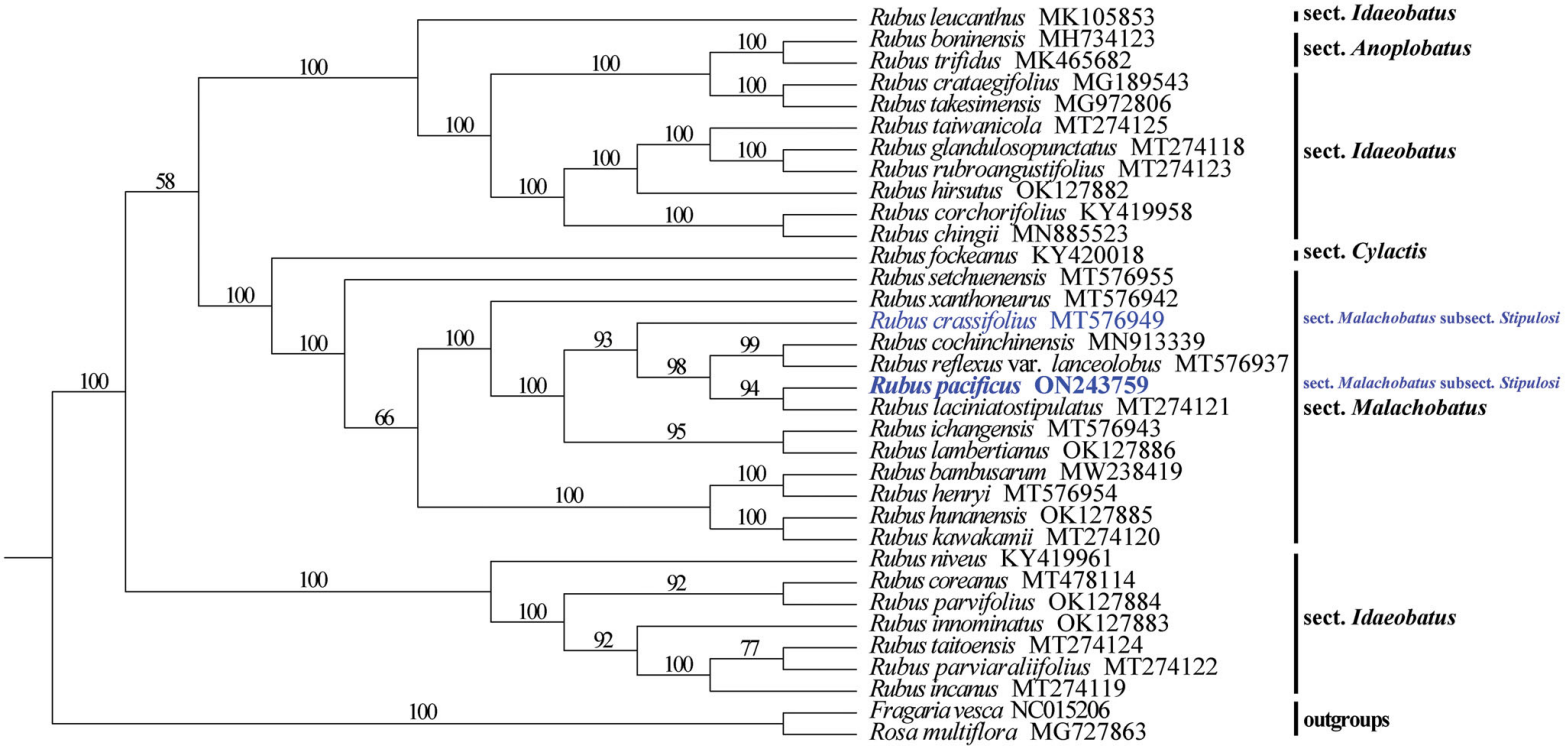
Maximum-likelihood (ML) phylogenetic tree based on complete chloroplast genomes of *Rubus pacificus*, 31 representative *Rubus* species and two outgroups. The bootstrap support values based on 1000 replicates are shown along the branches. The species belonging to *Rubus* sect. *Malachobatus* subsect. *Stipulosi* are marked in blue text.

## Data Availability

The genome sequence data that support the findings of this study are openly available in GenBank of NCBI at https://www.ncbi.nlm.nih.gov under the accession no. ON243759. The associated BioProject, SRA, and Bio-Sample numbers are PRJNA845828, SRR19542941 and SAMN28863898 respectively.
